# Barriers and Facilitators to the Implementation of eHealth Services: Systematic Literature Analysis

**DOI:** 10.2196/14197

**Published:** 2019-11-22

**Authors:** Björn Schreiweis, Monika Pobiruchin, Veronika Strotbaum, Julian Suleder, Martin Wiesner, Björn Bergh

**Affiliations:** 1 Institute for Medical Informatics and Statistics University Hospital Schleswig-Holstein and Kiel University Kiel Germany; 2 Consumer Health Informatics Special Interest Group German Association for Medical Informatics, Biometry and Epidemiology eV Cologne Germany; 3 GECKO Institute for Medicine, Informatics and Economics Heilbronn University Heilbronn Germany; 4 Zentrum für Telematik und Telemedizin GmbH Bochum Germany; 5 ERNW Research GmbH Heidelberg Germany; 6 Department of Medical Informatics Heilbronn University Heilbronn Germany

**Keywords:** eHealth, health information interoperability, policy, software design

## Abstract

**Background:**

The field of eHealth has a history of more than 20 years. During that time, many different eHealth services were developed. However, factors influencing the adoption of such services were seldom the main focus of analyses. For this reason, organizations adopting and implementing eHealth services seem not to be fully aware of the barriers and facilitators influencing the integration of eHealth services into routine care.

**Objective:**

The objective of this work is to provide (1) a comprehensive list of relevant barriers to be considered and (2) a list of facilitators or success factors to help in planning and implementing successful eHealth services.

**Methods:**

For this study, a twofold approach was applied. First, we gathered experts’ current opinions on facilitators and barriers in implementing eHealth services via expert discussions at two health informatics conferences held in Europe. Second, we conducted a systematic literature analysis concerning the barriers and facilitators for the implementation of eHealth services. Finally, we merged the results of the expert discussions with those of the systematic literature analysis.

**Results:**

Both expert discussions (23 and 10 experts, respectively) identified 15 barriers and 31 facilitators, whereas 76 barriers and 268 facilitators were found in 38 of the initial 56 articles published from 12 different countries. For the analyzed publications, the count of distinct barriers reported ranged from 0 to 40 (mean 10.24, SD 8.87, median 8). Likewise, between 0 and 48 facilitators were mentioned in the literature (mean 9.18, SD 9.33, median 6). The combination of both sources resulted in 77 barriers and 292 facilitators for the adoption and implementation of eHealth services.

**Conclusions:**

This work contributes a comprehensive list of barriers and facilitators for the implementation and adoption of eHealth services. Addressing barriers early, and leveraging facilitators during the implementation, can help create eHealth services that better meet the needs of users and provide higher benefits for patients and caregivers.

## Introduction

### Background

In 1999, the term *eHealth* was coined. The first publications defined it as a “new term needed to describe the combined use of electronic communication and information technology in the health sector. The use in the health sector of digital data—transmitted, stored, and retrieved electronically—for clinical, educational, and administrative purposes, both at the local site and at a distance” [1].

With the evolution of an increasing number of e-services, health care is providing many different eHealth services. In general, eHealth is associated with a positive influence on health care outcomes [2]. Improved cost-effectiveness, more information on a patient’s health status, and better communication between health care professionals are just some examples of the benefits of eHealth services [3,4]. However, there is no consistent picture of eHealth services’ adoption and broad acceptance. Often, eHealth services are not adopted and lack acceptance by their users [5]. However, for several services, domains, or patient groups, the levels of acceptance and related adoption rates are reported to be higher [6].

For several years, health care institutions have evaluated and started to use eHealth services to support patient care. The evolution of mobile phones and the broad availability of apps for prevention, wellness, and fitness scenarios has resulted in an increased importance of eHealth for the health care industry because these new services help to better support care processes [7,8]. Both the primary and secondary health care markets are important when it comes to eHealth services. With eHealth being an important economic factor, member countries of the Organisation for Economic Co-operation and Development spend an average of 8.9% of their gross domestic product on health [9]. In addition, start-ups and “big players” (eg, Google, Apple, Facebook, Amazon, and Microsoft) [10] play an important role in the eHealth economy. These economic factors drive changes in eHealth legislation in national health care systems, such as the eHealth Act in Germany and the Electronic Patient Record Act in Switzerland [11].

Several models are available to evaluate the use of technology (eg, Technology Acceptance Model [12] and Unified Theory of Acceptance and Use of Technology [13]), which are often adapted for evaluation in the eHealth domain [14,15]. Such models provide criteria for the evaluation of technology acceptance. The use of eHealth in routine care can be explained to a certain extent by these models [14-16]. However, those models may benefit from several additions and modifications, especially in relation to implementation.

Several reviews and projects have identified barriers and facilitators for eHealth service adoption in certain environments and disease contexts, such as mental health [17-19], veterans health care [20-22], and hypertension [23-25]. However, to the best of our knowledge, no overview, meta-analysis, or comprehensive list of barriers and facilitators affecting the adoption of eHealth services has been conducted and published.

For this reason, we organized two expert workshops and related discussion rounds to obtain an overview of the barriers and facilitators for the adoption of eHealth services. Both workshops were independent of a specific scenario and were accompanied by an exhaustive literature analysis.

### Objective

The objective of this work is twofold: to provide (1) a comprehensive list of barriers to be considered, and (2) a list of facilitators or success factors to help in planning and implementing eHealth services. It is not within the scope of this paper to provide another model for evaluating eHealth or telemedicine services.

## Methods

### Overview

Two different approaches were combined in this study. First, we wanted to obtain international experts’ current opinions on facilitators and barriers toward the implementation of eHealth services. This step helped to identify immediate experiences and knowledge bases especially from experts from countries with a higher level of digitization in health care. Second, we were interested in facilitators and barriers for implementation in completed projects and initiatives. Thus, two rounds of expert discussions at health informatics conferences in Europe were organized and held. Finally, a systematic literature analysis on barriers and facilitators for the implementation and adoption of eHealth applications was conducted.

### Expert Discussions

Two expert discussions were organized at conferences in Europe: (1) Medical Informatics Europe (MIE) 2015 in Madrid, Spain, and (2) eHealth Innovation Days (eHID) 2017 in Flensburg, Germany.

The MIE 2015 expert discussion in Madrid included 23 international experts from the field of medical and health informatics (15 participants from Europe; 5 from the Middle East, Asia, and America; and 3 German organizers). The primary topic of the expert discussion was “Consumer Health Informatics: Barriers and Facilitators of eHealth Usage Among Consumers.” The discussion included three short introduction talks, followed by three discussion groups on barriers for eHealth use among consumers [26]. However, due to time constraints resulting from the workshop format, the discussion mostly focused on barriers. Each group separately discussed the barriers to the use of eHealth applications and wrote them down on prompt cards. Once the discussions of five small groups were finished, each group presented their results briefly. The organizers of the expert discussion collected and aggregated the results in the format of a short workshop report [26].

The second expert discussion, on the topic of “Success Factors for Consumer-Centered eHealth Services,” was held in Flensburg, Germany. Participants were experts in the fields of medical and health informatics located in the Baltic Sea region, especially Sweden, Finland, Estonia, and Germany. An introduction followed by two short keynote talks constituted a starting point for the experts changing their perspective to one of five stakeholder groups. There were stakeholder groups for (1) citizens, patients, and family members (3 experts); (2) start-ups and application developers (4 experts); (3) researchers (3 experts); (4) policy makers and politicians (0 experts); and (5) data privacy officers and chief information officers (CIOs) (0 experts). The stakeholder groups for policy makers and politicians, as well as data privacy officers and CIOs were planned but were called off (0 participants). Each stakeholder group brainstormed on the success factors and facilitators for consumer-centric eHealth application use and/or its implementation. Next, each group presented briefly, and all groups discussed the results in a panel format. The results of each group were collected via flipcharts and consolidated by the authors in similar formats as the results of the first expert workshop held during MIE 2015.

### Literature Analysis

To identify relevant articles in the field, a PubMed search was conducted on May 28, 2018, including the following query terms: ((“telemedicine”[MeSH Terms] OR “telemedicine”[All Fields] OR “ehealth”[All Fields]) AND (“adoption”[MeSH Terms] OR “adoption”[All Fields]) AND barriers[All Fields] AND facilitators[All Fields]) AND ((“patients”[MeSH Terms] OR “patients”[All Fields]) OR consumers[All Fields]).

The time frame for potentially relevant articles was only limited by the search date. All articles published before this retrieval date were considered relevant. The resulting literature was filtered by scanning for actual mentions of barriers or facilitators for the adoption or implementation of any kind of eHealth application (see Textbox 1). In this context, titles, abstracts, and full-text articles were read to determine whether the article met the aforementioned criteria. For all identified papers, barriers and facilitators were extracted manually by one of the authors. Barriers and facilitators were listed in an Excel spreadsheet (see Multimedia Appendix 1). Next, a categorization was applied creating a mind map for barriers and facilitators separately (see Multimedia Appendix 2). This categorization was based on the three main categories as identified by Griebel et al [26]: (1) individual, (2) environmental and organizational, and (3) technical.

**Criteria for the inclusion and exclusion criteria of literature in the analysis.**

**Inclusion criteria**

- Published and listed on PubMed as of May 28, 2018
- Listing barriers for the implementation or adoption of eHealth services and/or listing success factors/facilitators for the implementation or adoption of eHealth services
- Articles in English and German

**Exclusion criteria**

- Article about research protocols of a planned study (ie, no results on barriers and/or facilitators)
- Abstract not available
- Article not about eHealth services
- Full text not accessible

### Comparison of Expert Discussions and Literature Analysis

One expert in the field of medical informatics categorized the barriers identified in the literature according to the categories provided by the study of Griebel et al [26] and extended the original mind map with the results from the literature analysis conducted for this study. The success factors for eHealth service adoption identified in the literature were categorized using the main categories (individual, environmental and organizational, and technical) in accordance with the categorization of barriers. The subcategorization of the results of the expert discussions refining the three categories was done where applicable and subsequently reviewed by a coauthor. The mind map, originally generated with results from the expert discussion on success factors, was then augmented with items found in the literature. Finally, the aggregated results of the Griebel et al study [26] were extended with the results from the expert workshop on success factors, with facilitators found in the literature, and displayed in a hierarchical form (mind map).

## Results

Results of the findings of the expert discussions are outlined first, followed by the results from the literature review. Both result sets are then compared for common and different attributes.

### Expert Discussions

**Figure 1:**
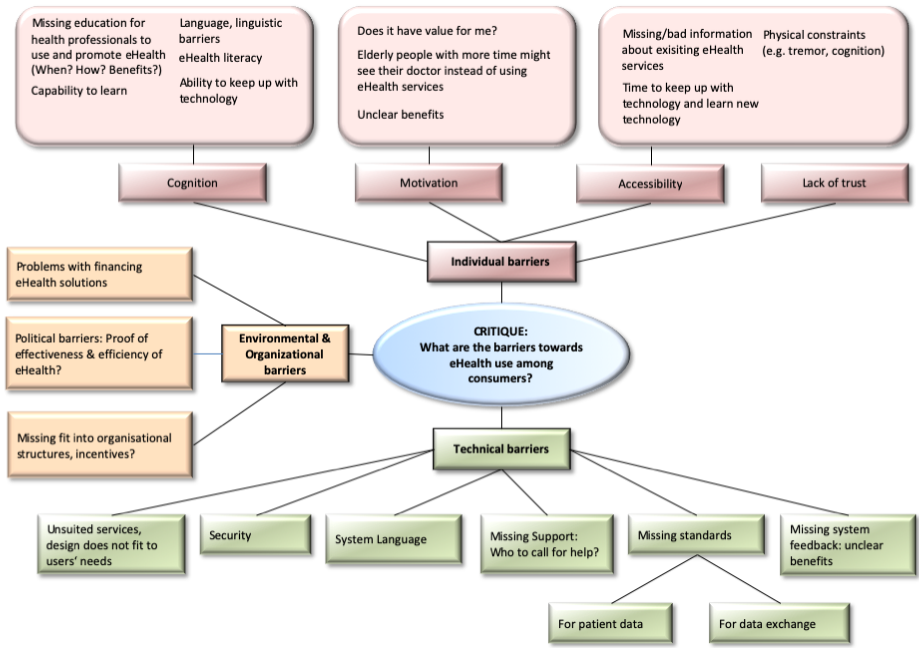
Barriers of eHealth usage among consumers identified in the first expert discussion at MIE 2015.

The expert discussion concerning barriers for eHealth services resulted in three categories of barriers: (1) individual, (2) environmental and organizational, and (3) technical barriers (see Figure 1). The category of individual barriers aggregated cognitive, motivational, accessibility, and trust-related barriers of individual consumers. Financial issues, political barriers, and organizational structures formed the category of environmental and organizational barriers. Unsuited services or design not fitting to the users’ needs were among the technical barriers. Security concerns were another barrier because often systems and network-enabled medical devices fail to provide an acceptable level of security. Additionally, system language, missing support (who to call for help?), missing standards (both for patient data and for data exchange), and missing system feedback leading to unclear benefits were mentioned as barriers for eHealth services.

**Figure 2:**
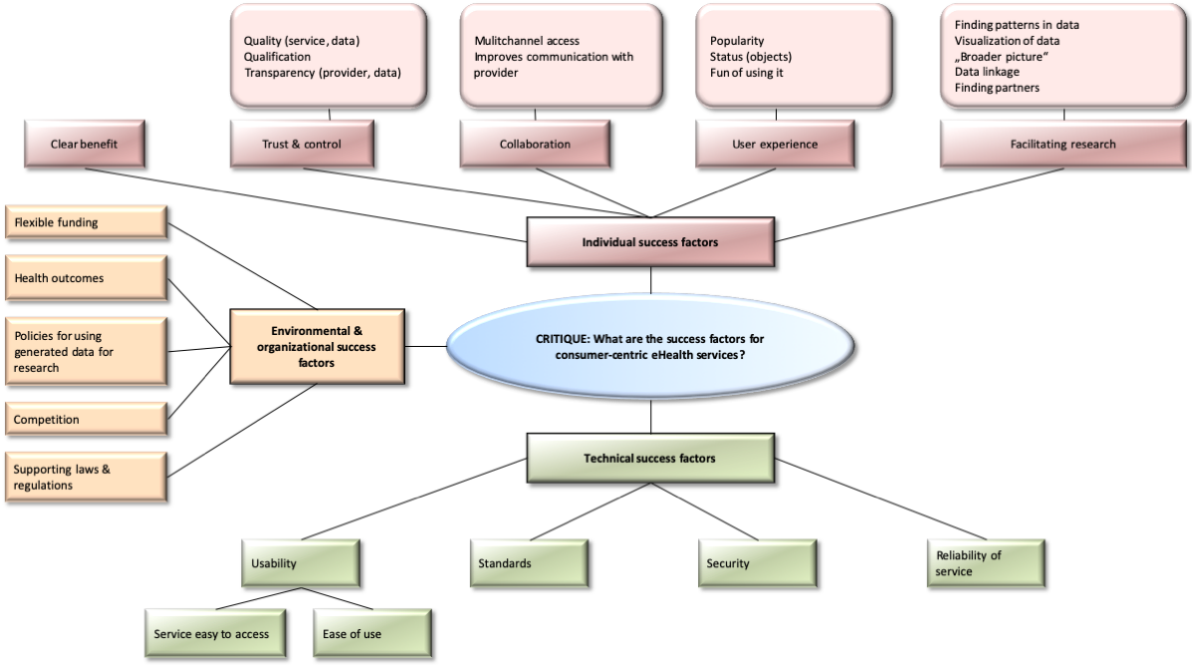
Success factors for consumer-centric eHealth services identified in the second expert discussion at eHID.

The expert discussion focusing on success factors and facilitators of consumer-centric eHealth services resulted in similar categories (individual success factors, environmental success factors, and technical success factors) (see Figure 2). We identified 31 success factors in the expert discussions. Subcategories of the individual success factors were a clear benefit of the service, trusting and controlling the service, the collaboration via the service, the service’s user experience, and that the service facilitates research. Flexible funding, health outcomes, policies for using generated data for research, competition, and supporting laws and regulations were the subcategories of environmental success factors. Usability, standards, security, and reliability of the service were subcategories of technical success factors.

### Literature Analysis

**Figure 3:**
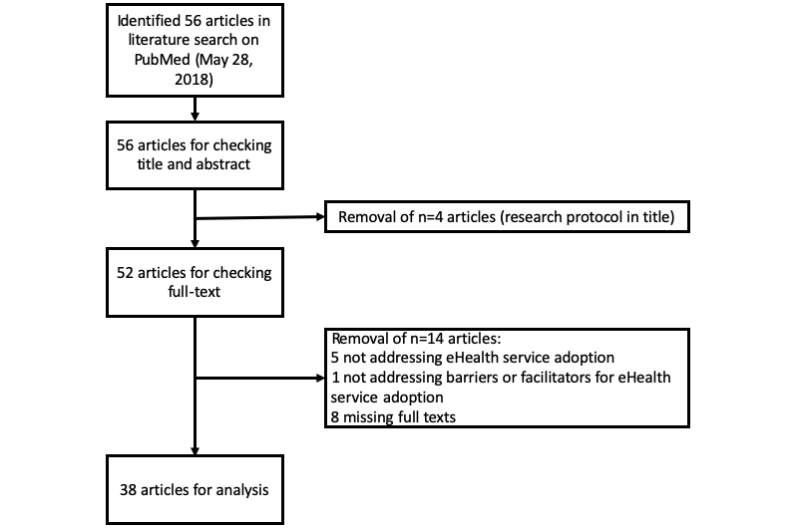
Flowchart for the identification of articles meeting the inclusion or exclusion criteria (see Textbox 1).

The literature analysis resulted in 56 publications published between December 27, 2007, and May 3, 2018. Of these publications, 38 were found to be relevant with full texts accessible to the authors for in-depth analyses [17-25,27-55] (see Figure 3). For the excluded 18 publications, either the full text was not accessible to the authors (n=8) or the articles did not describe, analyze, or present results about barriers or facilitators for the use of eHealth applications (n=10) (exclusion criteria see Textbox 1).

**Figure 4:**
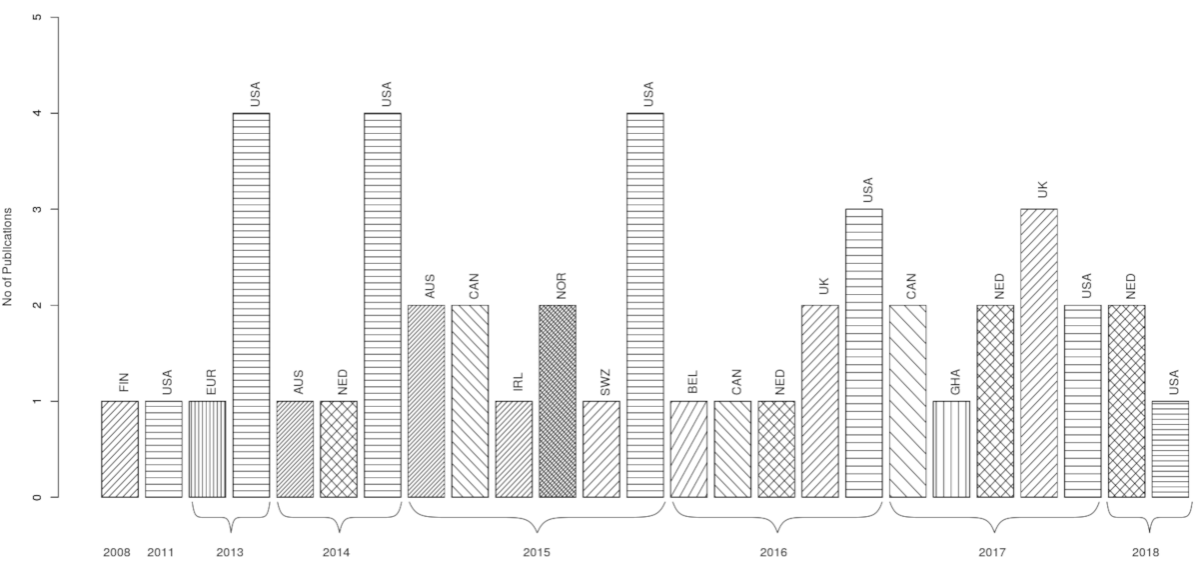
Included publications (before removal of missing full texts) by year and geographical location.

Publications, including the ones missing full text (n=8), originated mostly from the United States (19/46, 41%, published 2011-2018), followed by the Netherlands (6/46, 13%, published 2014-2018), Canada (5/46, 10%, published 2015-2017), the United Kingdom (5/46, 10%, published 2016-2017), Australia (3/46, 6%, published 2014-2015), and Norway (2/46, 4.35%, published 2015). Ghana (published 2017), Belgium (published 2016), Ireland (published 2015), Swaziland (published 2015), Europe (published 2013), and Finland (published 2008) each had one publication in this literature analysis (1/46, 2%; see Figure 4).

We identified 76 distinct barriers (33 individual, 25 environmental, and 18 technical) and a total of 268 facilitators (131 individual, 101 environmental, and 36 technical) in the literature (see Multimedia Appendix 1). The most frequent barrier in the literature was limited exposure/knowledge of eHealth (ie, poor digital health literacy) with 16 references [17,19-22,32,33,36,38,40,41,43,47,48,51,55], followed by 15 references of lack of necessary devices [19,20,22,24,32,37, 38,40,41,43,44,48,51,53,55], and problems with financing eHealth solutions [17-19,21,24,27,28, 33,38,39,42,47]. For a complete list of the top 10 barriers, see Table 1.

Ease of use was the most stated success factor found in the literature with seven references [20,30,33,43,44,47,53], followed by improves communication [23,28,35,44,55]. For a list of the top six facilitators, see Table 2.

**Table 1:** List of top 10 barriers mentioned in the literature.

Position	Perceived barrier	Mentions, n	References
1	Limited exposure/knowledge of eHealth (eg, poor digital health literacy)	16	[17,19-22,32,33,36,38,40,41,43,47,48,51,55]
2	Lack of necessary devices	15	[19,20,22,24,32,37,38,40,41,43,44,48,51,53,55]
2	Problems with financing eHealth solutions	15	[17-19,21,24,27,28,33,38,39,42,47]
4	Cognition	13	[19,23,24,30,36-38,44,47,51-54]
4	Security	13	[17,24,28,32-35,38-40,43,51,55]
6	Motivation	12	[17,23,24,36,39-41,43-45,53,55]
7	Accessibility	10	[23,36,38,39,43,46,47,51,53,55]
8	Unsuited services, design does not fit users’ needs	9	[24,33,35,38,43,46,47,52,53]
8	Confidentiality	9	[17,23,30-34,40,51]
10	Missing fit into organizational structures, incentives	8	[18,24,25,27,28,42,45,47]
10	Added workload	8	[23,24,28,29,38,39,44,54]

**Table 2:** List of the top six facilitators mentioned in literature^a^.

Position	Perceived facilitator	Mentions, n	References
1	Ease of use	7	[20,30,33,43,44,47,53]
2	Improves communication	5	[23,28,35,44,55]
2	Motivation	5	[23,36,47,52,53]
2	Integrated into care	5	[24,25,28,33,35]
5	Involvement of all relevant stakeholders	4	[18,25,47,54]
6	Availability of resources	3	[19,25,54]
6	User-friendliness	3	[27,36,39]

^a^ There were too many facilitators mentioned twice in the literature to list them all because it would make the table too long and difficult to read.

Only one of the included articles did not list any barriers [49], whereas three articles did not identify any facilitators [17,40,48]. For the analyzed publications, the count of distinct barriers reported ranged from 0 to 40 [24] (mean 10.24, SD 8.87, median 8). Likewise, between 0 and 48 facilitators [24] were mentioned in the literature (mean 9.18, SD 9.33, median 6).

### Comparison of Expert Discussions and Literature Analysis

The combination of the expert discussions (15 barriers) and literature analysis (n=76) yielded a total of 77 specific barriers. In sum, 292 facilitators or success factors were found during the expert discussions (n=31) and via the literature analysis (n=268).

All barriers identified in the literature could be matched to the main categories (individual barriers, environmental barriers, technical barriers) defined in the Griebel et al study [26]. The technical barriers category was also mentioned in Mileski et al [24]. In mapping barriers from the expert discussions and literature analysis, we found that all but one barrier resulting from the expert discussions—system language (ie, the language of the service in use, such as German or English)—were also covered by the literature.

Facilitators derived from the literature could also be mapped completely to the adapted main categories from Griebel et al [26].

## Discussion

### Principal Results

Several references in the literature report specific barriers of eHealth adoption and implementation (eg, missing eHealth strategies [19,33,38]). However, distinct success factors are only included in one or two references (eg, clear governance of national eHealth strategy [32]).

Although 24 success factors from the expert discussions were not included in the results of our literature analysis, only one barrier identified in the expert discussions (system language) was not found via the literature analysis in this study. Thus, the overlap of success factors between the literature analysis and expert discussions was smaller than for barriers.

The top 10 barriers (see Table 1) and top six facilitators (see Table 2) as identified by their mention in the literature analysis can be named important factors influencing the implementation and adoption of eHealth services. The remaining factors seem to be more specific to certain stakeholders or areas of application since eHealth is a large field.

We identified many more success factors than barriers for the adoption of eHealth services. One reason behind this finding might be that success factors are outlined to a greater extent and in higher detail compared with barriers, which are reported very coarse-grained. Publication bias could be another reason. Unsuccessful projects tended not to analyze the reasons for failure, or at least not to publish their insights, compared with successful projects. For example, the Good eHealth Report [56] lists lessons learned, but the case studies published are only successful ones. For example, the reasons for the delay of the German Telematics Infrastructure and services are not published in scientific studies at all.

### Limitations

The expert workshops were held only in Europe, which might have led to an underrepresentation of American, African, and Asian input to the discussions. Apart from PubMed, no further literature databases were consulted for this study. In addition, only one search with several parameters was conducted. However, the search parameters were adjusted several times to allow for more relevant articles to be found. Therefore, articles were randomly checked in the process. The literature analysis was restricted by search parameters including “barriers”’ and “facilitators” as well as “adoption” and “implementation,” which resulted in fewer articles found in the initial search. This also led to exclusion of fewer articles from the resulting list of publications because of irrelevance according to the chosen inclusion and exclusion criteria. Moreover, no white papers or reports by governments or other organizations were considered. Blog posts and articles by security professionals or operators and developers of these services, for example, were not included. A more comprehensive investigation with a focus on the aforementioned roles could consider these sources as well.

The categorization of barriers and facilitators was done by only one of the authors based on Griebel et al [26]. Thus, interrater reliability cannot be presented.

### Comparison With Prior Work

The literature analysis included several systematic reviews conducted by other researchers. However, these reviews were either focused on a specific eHealth application, disease, or patient subgroup. Kruse et al [20], for example, reviewed articles limited to military veterans with posttraumatic stress disorder to find out about factors that would influence telemedicine adoption. Mileski et al [24] focused their review on telemedicine for the self-management of hypertension. The systematic review by Ross et al [57] was limited to systematic reviews on factors influencing the implementation of eHealth published between 2009 and 2014. Ross et al searched with “MEDLINE, EMBASE, CINAHL, PsychINFO, and the Cochrane Library”—different databases than this study—so the relevant systematic reviews included in their study are only to a limited extent part of our literature analysis because we only searched PubMed. Ross et al used different categories for factors influencing the implementation of eHealth services informed by the Consolidated Framework for Implementation Research: innovation characteristics, outer setting, inner setting, characteristics of individuals, and process. Within these categories, Ross et al included components from all our top-level categories (individual, environmental and organizational, technical), such as adaptability and complexity (technical), and cost (environmental and organizational). Outer and inner setting, as described by Ross et al, would be included in environmental and organizational in our classification. However, Ross et al found “access to knowledge and information” to be a component of inner setting, which was added as an individual barrier (limited exposure/knowledge of eHealth) in our analysis. Another systematic review by O’Connor et al [43] analyzed qualitative studies to understand the factors affecting engagement with and recruitment to the use of eHealth applications. Bush et al [30] limited their systematic review to the pediatric population and the application type patient portal. The adoption of mHealth by health care professionals was the topic of the systematic review of Gagnon et al [33]. A narrative meta-review on e-mental health services was done by Batterham et al [17]. De Lusignan et al [32] did a literature review including electronic health records and patient access to health information, although eHealth applications were narrowed down to a subgroup.

In contrast to the studies included in our literature analysis, which were either based on literature analysis or reviews or experiences, we combined both expert discussions (experts' experience) and literature analysis. However, 24 success factors and one barrier from the expert discussions were not found in the literature. Also, the discussion groups “policy makers and politicians” and “data privacy officers and CIOs” could not be held due to a lack of participants.

Further approaches analyzed the applicability of the Technology Acceptance Model [13] and Unified Theory of Acceptance and Use of Technology [14,15] for the evaluation of eHealth services. However, these studies focused on contributing models for the evaluation of either eHealth services in general or a specific eHealth service instead of trying to provide a complete list of factors influencing their adoption. Models reflect only on certain details; they do not provide a holistic view of the impact factors for eHealth services.

Prior work includes analyses limited to within Europe, such as the Good eHealth Report [56] and MethoTelemed project [58]. The success factors given by the Good eHealth Report [56] are covered in the results of our literature analysis and expert discussions. Black et al [59] indicate that realizing the benefits of eHealth for quality and safety of health care is not guaranteed. They propose that more evaluation is necessary to identify all factors influencing eHealth services. The MethoTelemed project aimed to contribute to the evidence base on the impacts, benefits, and costs concerning telemedicine [58]. However, the project was constrained to telemedicine and focused mostly on methodological improvements.

In summary, the literature analysis conducted for this study, combined with findings from previous expert discussions, led to a more comprehensive list of barriers and facilitators for the adoption and implementation of eHealth services in general.

### Conclusions

This work provides a comprehensive list of barriers and success factors based on two expert discussions and a literature analysis (see Multimedia Appendix 2). This list allows different stakeholders to address barriers and make use of facilitators in the planning phase of eHealth services. Thus, our work provides a valuable resource for health professionals, researchers, health care institutions, or consumers. With this resource, these groups might create better-suited applications and thus raise the adoption levels of consumer-centric eHealth services. Further studies on missing publications regarding the number of unsuccessful projects and eHealth services are necessary to research publication bias in this field.

## Appendix

Multimedia Appendix 1 List of all 56 analyzed publications including extracted barriers and facilitators.

Multimedia Appendix 2 Mindmap of all barriers and facilitators identified in expert discussions and literature.

## Abbreviations

CIO: chief information officer
eHID: eHealth Innovation Days Conference
MIE: Medical Informatics Europe Conference
