# Supplementary figures and images for: Barriers and Facilitators to the Implementation of eHealth Services: Systematic Literature Analysis
Source: J Med Internet Res. 2019 Nov 22;21(11):e14197. doi: 10.2196/14197 (PMC6898891; doi:10.2196/14197)

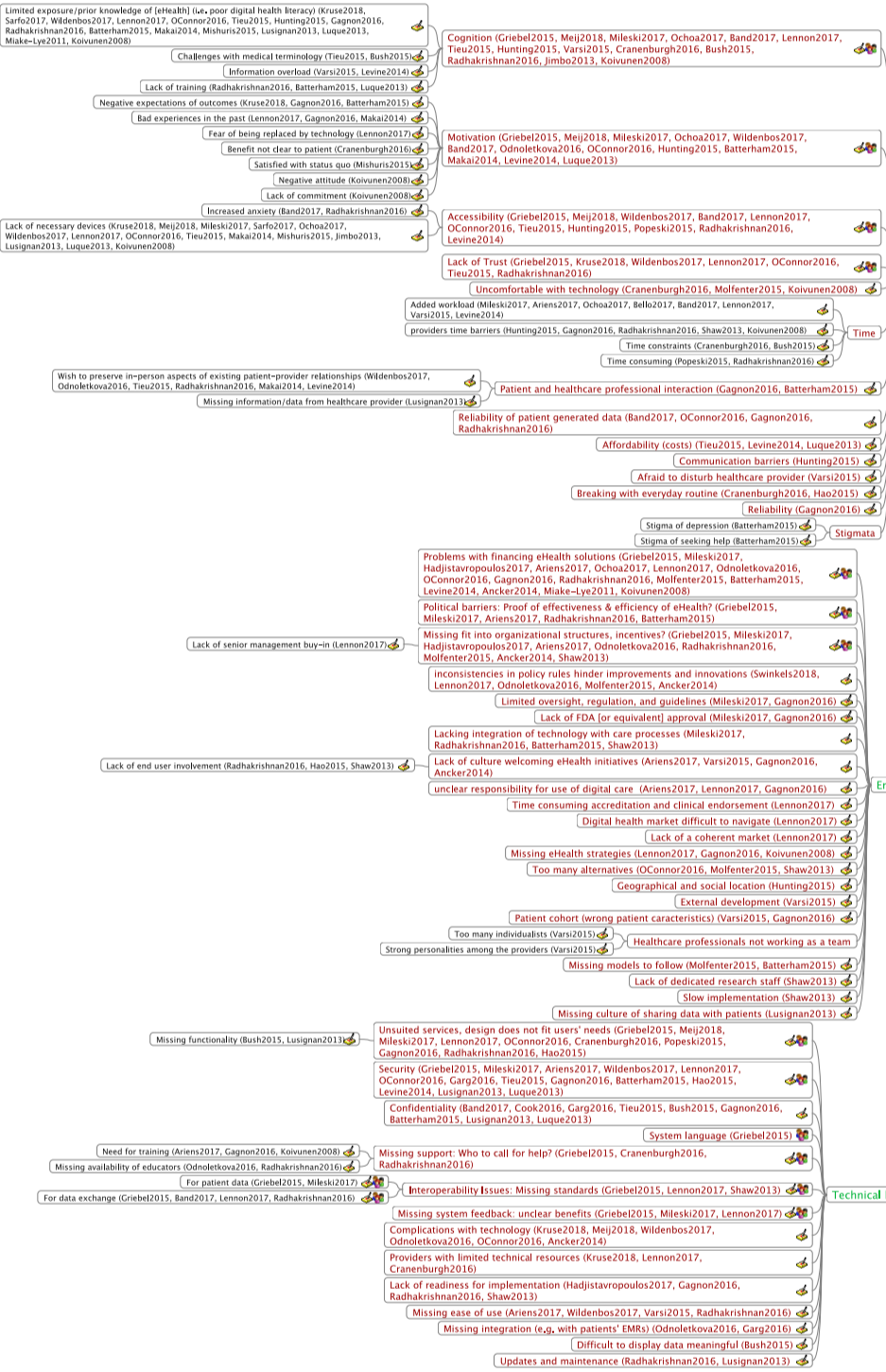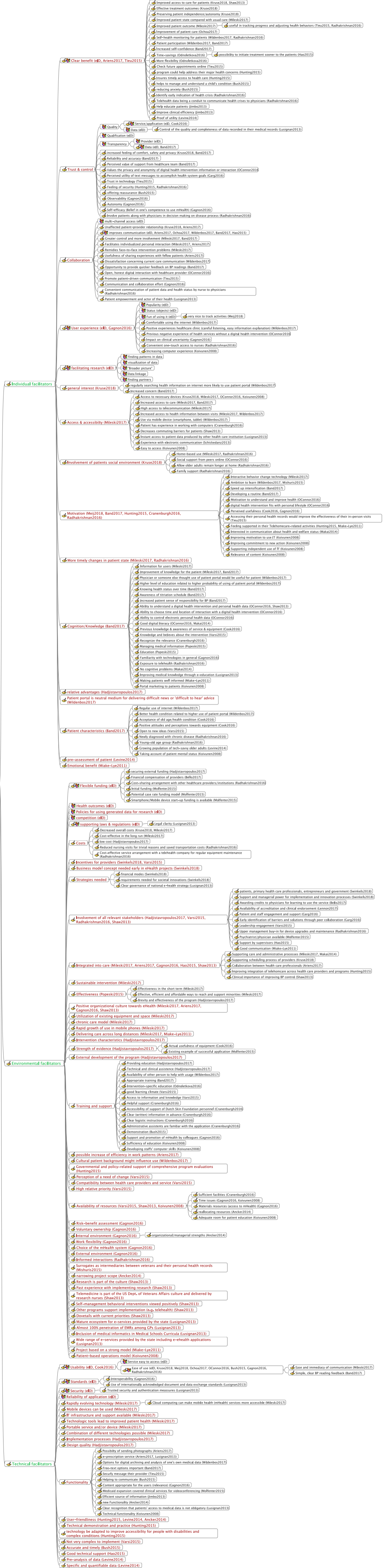

Supplement: Multimedia Appendix 2 [file jmir_v21i11e14197_app2.pdf]
